# Optimization of exogenous CeO_2_ nanoparticles on Pak choi (*Brassica rapa* L. var. chinensis) to alleviate arsenic stress

**DOI:** 10.3389/fpls.2024.1497926

**Published:** 2025-01-17

**Authors:** Rohina Tabassam, Shoaib Ahmad, Adiba Khan Sehrish, Azeem Ahmad, Sarah Owdah Alomrani, Abdul Ghafoor, Tahira Akram, Muhammad Ali Alshehri, Sumaira Noor, Shafaqat Ali

**Affiliations:** ^1^ State Key Laboratory of Pollution Control and Resource Reuse, School of the Environment, Nanjing University, Nanjing, Jiangsu, China; ^2^ Soil and Water Chemistry Laboratory, Institute of Soil and Environment Sciences, University of Agriculture, Faisalabad, Pakistan; ^3^ Department of Biology, College of Science and Arts, Najran University, Najran, Saudi Arabia; ^4^ Center for Water and Environmental Studies, King Faisal University, Al-Ahsa, Saudi Arabia; ^5^ Department of Biology, Faculty of Science, University of Tabuk, Tabuk, Saudi Arabia; ^6^ State Key Laboratory of Pharmaceutical Biotechnology, School of Life Sciences, Nanjing University, Nanjing, Jiangsu, China; ^7^ Department of Environmental Sciences, Government College University Faisalabad, Faisalabad, Pakistan; ^8^ Department of Biological Sciences and Technology, China Medical University, Taichung, Taiwan

**Keywords:** arsenic, CeO_2_ NPs, Pak choi, nutrient content, chlorophyll content, antioxidant enzyme activity

## Abstract

Arsenic (As) is a regulated hazardous substance that persists in the environment, causing issues related to environmental health, agriculture, and food safety. Cerium oxide nanoparticles (CeO_2_ NPs) are emerging sustainable solutions for alleviating heavy metal stress. However, their effectiveness and optimization for foliar application in reducing As stress, especially in Pak choi, has not been reported yet. Hence, this study aims to examine the effects of foliar application of CeO_2_ NPs (75,000,000, 150,000,000, and 300,000,000 ng/L) on the growth, nutrient availability, and antioxidant enzymatic activities of Pak choi plants under As stress. The findings showed that foliar application of 75,000,000 ng/L CeO_2_ NPs significantly increased shoot length (77.32%), root length (80.98%), and number of leaves (80.23%) as compared to control without NPs. The lowest dose of CeO_2_ NPs (75,000,000 ng/L) increased antioxidant enzyme activities such as peroxidase (86.10%), superoxide dismutase (81.48%), and catalase (52.07%), while significantly reducing malondialdehyde (44.02%), hydrogen peroxide (34.20%), and electrolyte leakage (43.53%). Furthermore, foliar application of 75,000,000 ng/L CeO_2_ NPs significantly increased the content of zinc (81.02%), copper (56.99%), iron (88.04%), manganese (68.37%), magnesium (76.83%), calcium (61.16%), and potassium (84.91%) in leaves when compared to control without NPs. The same trend was observed for shoot and root nutrient concentrations. Most importantly, 75,000,000 ng/L CeO_2_ NPs foliar application significantly reduced shoot As (45.11%) and root As (20.89%) concentration compared to control, providing a reassuring indication of their potential to reduce As concentration in plants. Our study’s findings are of utmost importance as they indicate that lower concentrations of foliar-applied CeO_2_ NPs can be more effective in enhancing crop nutrition and reducing heavy metals than higher concentrations. This article is intended to present critical issues of As contamination in agricultural soils, which imposes substantial risks to crop productivity and food security.

## Graphical Abstract

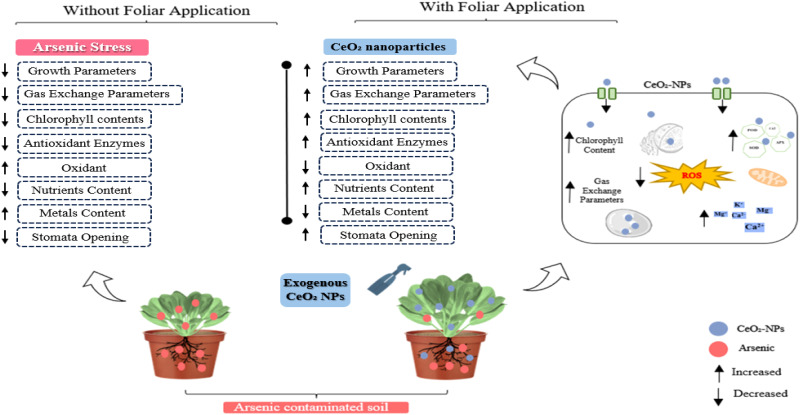

## 1 Introduction

Heavy metals (HMs) have become significant environmental toxins among abiotic stresses. Their presence in soil severely impacts food security due to negative effects on crop nutritional quality (Aborisade et al., 2023). Arsenic (As) is a toxic element that poses a significant threat to ecosystems and human health, particularly in its inorganic forms in soil (El Sharkawy et al., 2024). As enters the environment from natural sources (Neog et al., 2024) and human activities, such as waste discharge, ore mining, sewage irrigation, and excessive use of fertilizers and pesticides (Khatun et al., 2022). Higher concentrations of As can degrade soil properties, kill beneficial bacteria, and disrupt plant growth and development (Zhang et al., 2021), thereby affecting the food chain and the ecosystem (Angon et al., 2024). As contamination in the soil can lead to reduced growth, impaired photosynthetic activity, modulation in metabolic pathways, lowered nutrient uptake, and increased oxidative stress in plants (Nabi et al., 2021; Abbas et al., 2018; El Sharkawy et al., 2024). Plants exposed to high As concentrations generate reactive oxygen species (ROS), which disrupt cellular redox balance (Solórzano et al., 2020; Zulfiqar and Ashraf, 2022), damage cell membrane phospholipids, cause ion leakage, and lead to lipid peroxidation, ultimately reducing yield (Sachdev et al., 2021). To combat oxidative damage, plants activate enzymatic and non-enzymatic defense mechanisms to reduce ROS levels (Rajput et al., 2021). Several studies also reported As toxicity on various aspects of plant health. For instance, As was shown to affect photosynthetic activity in rice (Asgher et al., 2021) and *Brassica napus* (Bano et al., 2022), and morphological growth and antioxidant enzyme activities in wheat (Manzoor et al., 2023). As is classified as a Class I carcinogen by the International Agency for Research on Cancer (Duker et al., 2005) and raises major public health concerns due to its highly toxic, non-biodegradable nature, and tendency to accumulate in the body, potentially leading to cancer (Rehman et al., 2021). Consequently, developing innovative approaches to mitigate As contamination is crucial to address pressing environmental challenges and ensure food security and safety. Arsenic (As) exposure has raised global concerns due to its harmful effects on the food supply chain. Although various strategies are in place to reduce arsenic stress in plants, significant research gaps must be addressed.

Nanoparticles (NPs) have emerged as potential tools to reduce HM toxicity and to promote nutrient use efficiency and sustainability (Zhou et al., 2020). NP effectiveness in soil is limited due to aggregation with soil particles (Mishra et al., 2021). Therefore, foliar application may be a more effective way to enable NPs to enter plant tissues, reduce ROS, and minimize chemical inputs (Liu et al., 2022). For example, zinc oxide nanoparticles (ZnO NPs) applied as foliar treatment significantly alleviated As-induced oxidative stress in rice (Jalil et al., 2023). Silica nanoparticles (Si NPs) have also shown strength in coping As stress through As sequestration, which improves root hydraulic conductance and enhances antioxidant activity and membrane stability (David et al., 2024). Among various NPs, cerium oxide nanoparticles (CeO_2_ NPs) have enormous potential to improve plant productivity, stress resilience, and targeted nutrient availability (Mohammadi et al., 2021). In addition, CeO_2_ NPs have a high capacity for adsorbing hazardous HMs, including arsenic, lead, and cadmium (Rossi et al., 2018). Previous research indicated that CeO_2_ NPs can adsorb As on their surfaces, potentially reducing the bioavailability of As to plants (Neil et al., 2021). CeO_2_ NPs significantly enhanced the growth of sunflower plants, reduced oxidative stress, and improved enzymatic activities under chromium stress (Ma et al., 2022). Wang et al. (2018) found that CeO_2_ NPs enhanced growth metrics in rice by modifying arsenic speciation and decreasing arsenic accumulation in plant tissues. Lower concentrations of CeO_2_ NPs (10–100 mg/L) improved the growth, biomass, photosynthesis, and chlorophyll concentration of maize and peas and plant nutrient homeostasis (Gui et al., 2015; Ahmad et al., 2024). Moreover, lower concentrations of these NPs represent the higher activity of antioxidants in scavenging ROS, and protecting cellular processes, including photosynthetic activity, against oxidative stresses, thereby maintaining ionic homeostasis (Iftikhar et al., 2020). Salmen and Alharbi (2024) reported that low doses of CeO_2_ NPs in rice plants significantly reduced lead (Pb) concentration. Additionally, these treatments resulted in a notable increase in plant growth, biomass, gas exchange characteristics, antioxidant enzymatic activity, and an increase in other antioxidants, such as flavonoids, phenols, and prolines, while simultaneously reducing oxidative stress. In contrast, higher concentrations of these NPs showed retarded plant growth and inhibition of enzyme functions (Kamali-Andani et al., 2022). For example, CeO_2_ NPs (200 mg/kg) decreased the photosynthetic rate and CO_2_ assimilation efficiency of *Clarkia unguiculata*, likely by disrupting the energy transfer from photosystem II to the Calvin cycle (Conway et al., 2015).

Leafy vegetables generally absorb higher amounts of HMs compared to seeds and fruit-bearing vegetables largely due to their dense root systems (Alexander et al., 2006). However, As uptake in leafy vegetables like *Brassica rapa* L. remains poorly understood, limiting our knowledge of As bioavailability and safety assessments in these crops. Pak choi (*Brassica rapa* L.), a popular leafy vegetable, cultivated globally, is renowned for its high yield and nutritional values, providing essential minerals and vitamins (Hong and Gruda, 2020; Wang et al., 2022). Pak choi has the potential to accumulate harmful HMs like As, which can pose significant health risks (Dai et al., 2019). Addressing this concern, the present study is novel in exploring Pak choi’s morphological, physiological, and nutritional response to As toxicity. The objectives of this study were (i) to determine the effects of varying concentrations of CeO_2_ NPs (75,000,000, 150,000,000, and 300,000,000 ng/L) on plant growth parameters; (ii) to assess the physiological response of CeO_2_ NPs, including photosynthetic parameters, antioxidant enzyme activity, and stress indicators; and (iii) to determine the impact of varying concentrations of CeO_2_ NPs on nutrient uptake and availability in Pak choi. By evaluating the effect of different concentrations on plant growth, physiology, and nutritional quality, the ultimate goal of the present study was to identify the optimal concentration of CeO_2_ NPs for promoting plant growth and nutritional content while minimizing As uptake. This research will provide valuable insights into the safe and effective application of CeO_2_ NPs in agricultural practices under As-contaminated soil.

## 2 Materials and methods

### 2.1 Soil collection and analysis

The soil that was used for this experiment was taken from an area situated on River Ravi bank, Lahore, Punjab, Pakistan (31°32′14.0″N 74°14′18.0″E). Soil samples were randomly obtained using a stainless-steel spade, ranging in depth from 0 to 20 cm. The samples were well mixed to ensure homogeneity. The gathered samples were subsequently air-dried without being exposed to sunlight and filtered using a 2-mm sifter to guarantee uniform particle size. A sample of this soil was taken for analysis. For pre-soil characterization, including particle size, standard procedures were followed (Bouyouces, 1962). The soil pH and EC were measured following Rhoades' methods (1996). Organic matter analysis was conducted as described by Nelson and Sommers (1982), and arsenic was determined according to Suarez (1996). Detailed physiochemical properties of soil are shown in **Table 1**.

**Table 1:** The soil physiochemical properties used in this study.

Parameters	Units	Values
Texture	**–**	Sandy loam
pH	–	7.60
EC	dS/m	2.045
Organic matter	(%)	1.045
Nitrogen	(mg kg^−1^)	0.84 ± 0.54
Magnesium	(mg kg^−1^)	184.21 ± 20.08
Cupper	(mg kg^−1^)	1.17 ± 5.17
Calcium	(mg kg^−1^)	3.04 ± 22.84
Total As	(mg kg^−1^)	190 ± 0.12

### 2.2 Plant growth, treatment, and exposure

The pot experiment was carried out in a botanical garden in a natural environment with a temperature of 28/20°C day/night and a relative humidity 67% ± 5% in Government College University, Faisalabad, Pakistan, which had three replications using a completely randomized group design. Plastic pots were used (weight, 54 g; diameter, 14 cm; and height, 12 cm) containing 1 kg of soil polluted with arsenic. Seeds of the Pak choi (*Brassica rapa* L. var. chinensis) were purchased from the Ayub Agricultural Research Institute, Faisalabad, Punjab, Pakistan. Eight healthy and evenly sized Pak choi seeds were planted in each pot on 2024 February 28. The recommended N:P:K (25:12:8) potassium, phosphorus, and nitrogen doses were applied to avoid nutrient deficiency (Feng et al., 2013). After 15 days of planting, seedlings were thinned to three plants per pot. CeO_2_ NPs (99.9%, < 25 nm) were purchased from Sigma Chemical Company Limited (Shanghai, China). Tween-20 was used as an adhesive agent, and CeO_2_ NPs were sprayed at different intervals with levels of 75,000,000, 150,000,000, and 300,000,000 ng/L (Jahani et al., 2019; Liu et al., 2022) and seven foliar sprays were applied in total after 72-h intervals. No plants died during planting, and no additional agronomic measures such as pesticides were taken.

### 2.3 Growth assay

Carefully uprooted plants were gently washed with ultrapure water to get rid of any remaining soil deposits and dust. The length of the shoots and roots was measured using a graduated ruler, and the dry weight of roots and shoots was measured using an electrical balance after the plants had been dried for 48 h at 80°C in an oven (Model: 101-OAB Digital Lab Thermostatic Electric Incubator).

### 2.4 Photosynthesis exchange parameters and pigment assay

The chlorophyll content was measured as in Arnon (1949). Fresh leaves (0.5 g) were crushed with acetone (80% v/v) and the material was centrifuged at 4°C with a speed of 15,000 rpm to extract the chlorophyll content. The absorbance was measured using a Spectrophotometer (UV2350 UV–Vis spectrophotometer; Unico Shanghai Instrument Co., Ltd). at 470, 663, and 645 nm. Gas exchange parameters (LI-6800, Li-Cor Inc., Lincoln, Nebraska, USA) were recorded during the day, from 10:00 to 12:00 a.m. The specific measurement conditions of the photosynthetic meter are as follows: flow rate: 500 μmol s^−1^; pressure: 0.1 kPa; humidity: 50%–75%; mixing fan speed: 10,000 rpm; temperature: 25°C; light intensity: 1,200 μmol m^−2^ s^−1^; and CO_2_ flow rate: 400 μmol mol^−1^.

### 2.5 Determination of antioxidant enzymes and oxidant activity

After 4 weeks of germination, the antioxidant enzymatic activity was measured. A pre-chilled pestle and mortar were used to homogenize fresh leaves (0.3 g) in a 50 mM pH 7.8 phosphate buffer solution (PBS). The mixture was centrifuged (Fresco 17 microcentrifuge) at 4°C at a speed of 12,000 rpm for 15 min to get a supernatant (Khan et al., 2019). The collected supernatant was used to determine catalase (CAT), peroxidase (POD), ascorbate peroxidase (APX), and superoxide dismutase (SOD). The solution consisted of pH 7.8 phosphate buffer (50 mM), enzyme extract, and H_2_O_2_ (300 mM) for CAT estimation. The activity was measured using Aebi (1984) method, with the absorbance at 240 nm. A reaction solution comprising (75 µM) NBT, L-methionine (130 mM), riboflavin (20 µM), and EDTA-NA_2_ (100 µM) together with the enzyme extract was used to measure the SOD activity (Zhang et al., 2008). The spectrophotometer was used to determine the absorbance at a wavelength of 560 nm. By making a reaction solution with enzyme extract, H_2_O_2_ (300 mM), PBS, and ascorbic acid (7.5 mM), the APX activity was determined. The measurement was made for 30 and 60 s at 290 nm. The Chance and Maehly (1955) method were used to measure the activity of POD. Zhang and Kirkham (1994) used the method of determining the malondialdehyde (MDA) content. After centrifugation (4,800 rpm) to collect the supernatant, the reaction solution consisted of a mixture of ground TCA (0.5%) and TBA (5%) combined with enzyme extract and heated at 95°C. The absorbance at 632 and 532 was obtained to determine MDA content. Dionisio-Sese and Tobita (1998) measured electrolyte leakage (EL) in leaves, while Jana and Choudhari (1982) measured hydrogen peroxide (H_2_O_2_) in leaves.

### 2.6 Measurement of flavonoid content, proline content, and total phenolic content

The flavonoid content was estimated based on the methodology of Elzaawely and Tawata (2012). The screening process for leaf proline content was carried out by Bates et al. (1973). Plant leaf material (500 mg) was first chopped and ground into a smooth paste. After that, this paste was mixed with a 10-mL, 3% sulfosalicylic acid solution. After that, the solution was centrifuged for 15 min at 6,000 rpm. Following this process, the sample that had been prepared (2 mL) was put into a glass test tube with 2 mL of acid ninhydrin and glacial acetic acid (2 mL). The test tube was then placed in a hot water bath for 60 min. Then, 4 mL of toluene was added to the mixture (2 mL), and the mixture was centrifuged for 5 min at 5,000 rpm. After 30 min, the upper layer was isolated using a separating funnel, and the absorbance of the sample at a wavelength of 520 nm was measured using a spectrophotometer. The total phenolic content of the sample was measured with the Folin-Ciocalteu methods. The Folin-Ciocalteu method was followed with some modifications (Singleton et al., 1999).

### 2.7 Observation of leaves stomata by scanning electron microscopy

The scanning electron microscopy (SEM) technique followed the described method (Salehi et al., 2021). In an ethanol series (30%, 50%, 70%, 80%, 90%, 95%, and 100%), the leaves were dehydrated after being fixed in 2.5% (w/v) glutaraldehyde for 4 h. A critical point dryer was used to dry the samples. A Hitachi E-1010 sputter coater was used to apply gold to the materials, and an SEM (Hitachi Model S-3400N) was used to assess the results.

### 2.8 Nutrient profiling and arsenic concentration assay

The 0.1-g dried samples of plants from above- and belowground were digested using a hotplate at 160°C for 40 min in Teflon containers using a combination of 2 mL of pure HNO_3_ and 8 mL of H_2_O_2_ (v/v: 4:1) until the solution turned transparent. Then, As content and macro- and micronutrients were measured using ICP-OES (Huang et al., 2004).

### 2.9 Statistical analysis

The statistical analysis was evaluated using the SPSS software program (2020). Statistical analysis of collected data was processed by one-way analysis of variance (ANOVA) and Tukey’s honestly significant difference (HSD) *post-hoc* test was performed to evaluate significant differences and pairwise comparison among treatments. Different letters on the bars denote significant differences among treatments (*p* ≤ 0.05). OriginPro, Version 2023b (Origin Lab Corporation, Northampton, MA, USA) and Excel (Office 2019 for Windows) were used to plot graphs.

## 3 Results

### 3.1 Growth parameters response of Pak choi to the application of cerium oxide nanoparticles under arsenic stress

The present study achieved positive results by applying different CeO_2_ NPs in the foliar form under As-contaminated soil. The results depicted that As stress significantly minimized plant growth traits. Meanwhile, the foliar application of CeO_2_ NPs significantly enhanced the growth traits of Pak choi under As stress. Data regarding growth parameters such as length of root and shoot, fresh and dry weight, and number of leaves as affected by the application of CeO_2_ NPs are presented in **Table 2**. For shoot length, significant (*p* ≤ 0.05) improvements were observed under 75,000,000 ng/L CeO_2_ NPs (77.32%), 150,000,000 ng/L CeO_2_ NPs (59.50%), and 300,000,000 ng/L CeO_2_ NPs, (25.15%), compared with control plants. The improvement of root length from 80.98%, 63.45%, and 33.38% was achieved by applying 75,000,000 ng/L CeO_2_ NPs, 150,000,000 ng/L CeO_2_ NPs, and 300,000,000 ng/L CeO_2_ NPs treatments, respectively, compared with control. Similarly, 75,000,000 ng/L CeO_2_ NPs had a notable positive effect on shoot fresh/dry weight (85.94/86.17%) compared to higher levels and the control group. Higher levels of CeO_2_ NP application, such as 150,000,000 ng/L and 300,000,000 ng/L, also improved shoot fresh/dry weight (34.18/61.34%) and (34.18/31.56%), respectively. Comparing the root fresh/dry weights of the control group to the treated groups, CeO_2_ NPs showed better responses—74.19%/69.19%, 61.29/40.23%, and 29.03/15.40%—at the level of 75,000,000 ng/L, 150,000,000 ng/L, and 300,000,000 ng/L CeO_2_ NPs respectively. In terms of number of leaves, the higher-concentration 150,000,000 ng/L CeO_2_ NPs (56.22%), followed by 300,000,000 ng/L CeO_2_ NPs (32.20%), showed minimum improvement in number of leaves as compared with 75,000,000 ng/L CeO_2_ NPs (80.23%), which significantly (*p* ≤ 0.05) boosted number of leaves and all growth parameters under As stress, when compared to stressed control.

**Table 2:** Effect of CeO_2_ NPs on growth parameters of Pak choi (*Brassica rapa* L. var. chinensis) grown under arsenic-contaminated soil.

Growth parameters							
Treatments	Root length (cm)	Shoot length (cm)	Root fresh weight (g pot^−1^)	Root dry weight (g pot^−1^)	Shoot fresh weight (g pot^−1^)	Shoot dry weight (g pot^−1^)	No. of leaves (per plant)
NPs 0 (ng/L)	3.40 ± 0.47^c^	7.54 ± 0.89^b^	0.21 ± 0.031^c^	0.045 ± 0.0055^c^	3.13 ± 0.26^c^	0.94 ± 0.10^c^	4 ± 0.37^c^
NPs 75,000,000 (ng/L)	6.16 ± 0.42^a^	13.37 ± 0.98^a^	0.36 ± 0.024^a^	0.077 ± 0.0064^a^	5.82 ± 0.44^a^	1.75 ± 0.12^a^	8 ± 0.42^a^
NPs 150,000,000 (ng/L)	5.56 ± 0.59^ab^	12.03 ± 1.10^a^	0.33 ± 0.027^a^	0.063 ± 0.0082^ab^	5.06 ± 0.24^ab^	1.52 ± 0.11^a^	7 ± 0.54^ab^
NPs 300,000,000 (ng/L)	4.54 ± 0.62^bc^	9.44 ± 0.70^b^	0.27 ± 0.030^b^	0.052 ± 0.0057^bc^	4.20 ± 0.42^b^	1.24 ± 0.10^b^	6 ± 0.58^b^

Values are the means ± standard; deviation different letters indicate significant difference among treatments at *p* ≤ 0.05.

### 3.2 Effect of CeO_2_ NPs (foliar) on photosynthetic contents and gas exchange parameters in the Pak choi under As stress

**Figure 1:**
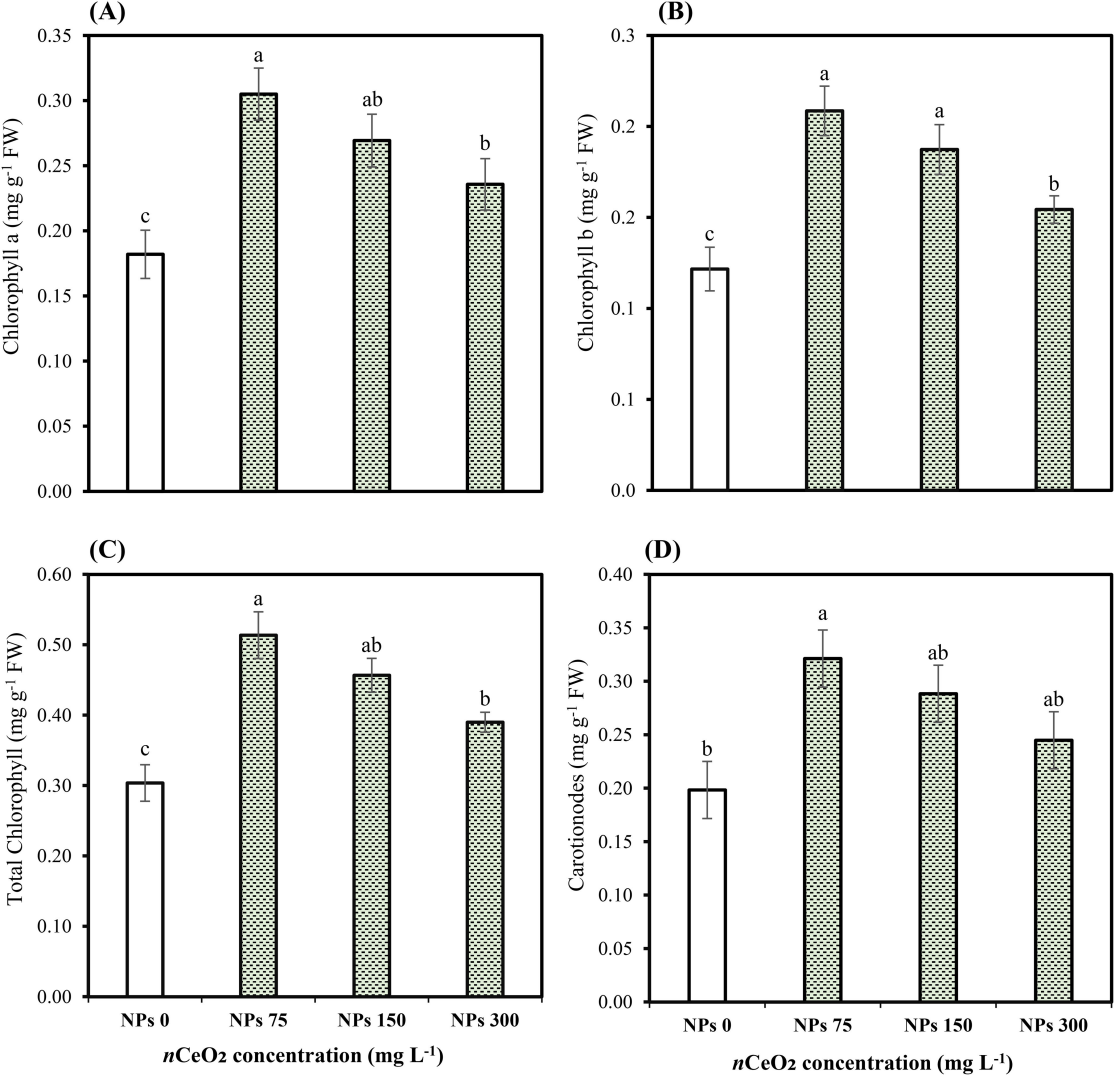
Effect of CeO2 NPs on chlorophyll a **(A)**, chlorophyll b **(B)**, total chlorophyll **(C)**, and carotenoids **(D)** of Pak choi (*Brassica rapa* L. var. chinensis) grown under arsenic-contaminated soil. Data are presented as the mean of three replicates ± standard deviation (SD). Different lowercase letters indicate significant/non-significant differences among treatments according to Tukey’s test (*p* ≤ 0.05).

**Figure 2:**
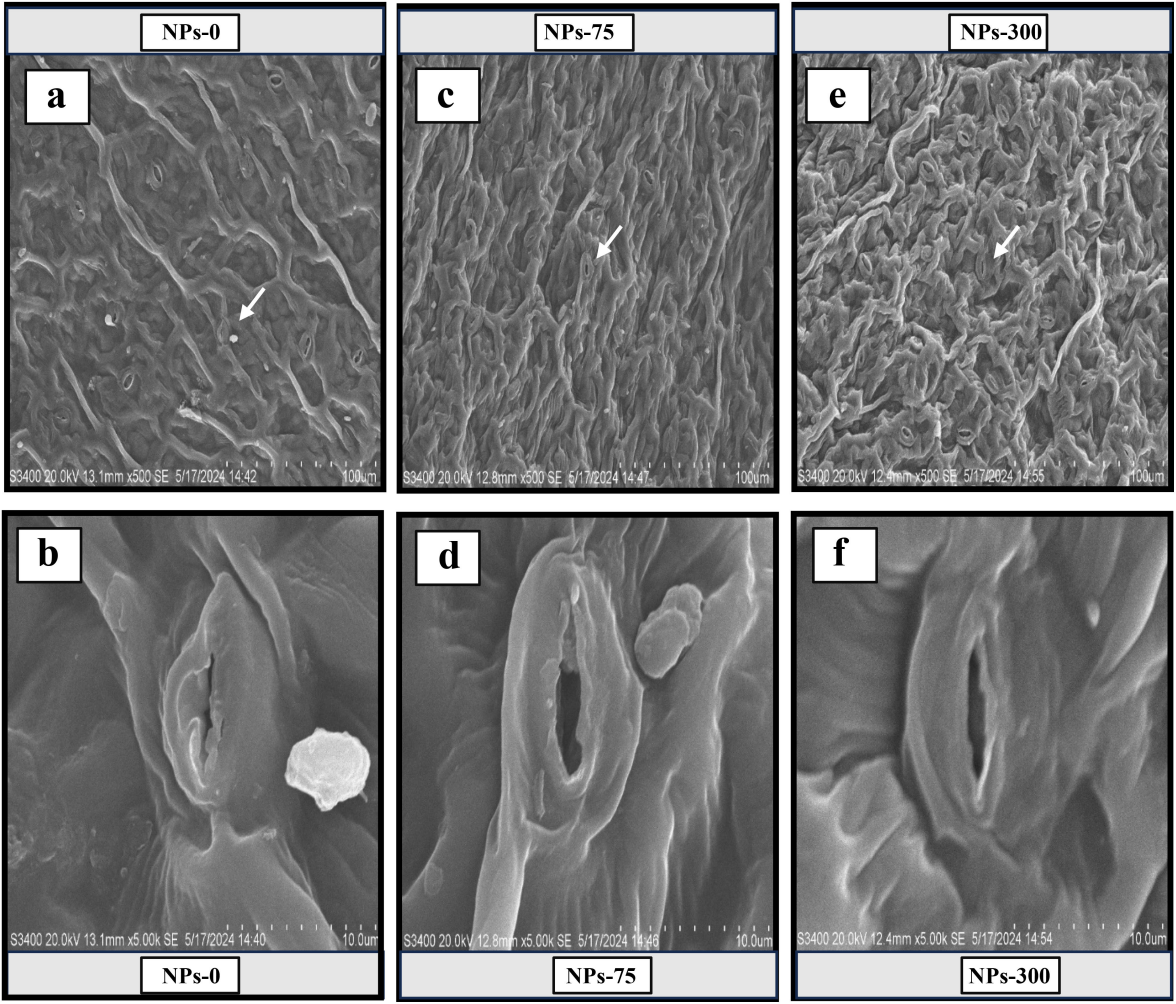
Scanning electron microscope (SEM) images of stomata showed the responses of foliar application cerium oxide nanoparticles (CeO_2_ NPs) on the stomatal aperture of Pak choi (*Brassica rapa* L. var. chinensis) leaves under arsenic stress. **(A, B)** revealed the complete closure of leaves stomata at 0 ng/L (CeO_2_ NPs) under maximum arsenic stress conditions. **(C, D)** showed full opening of leaves’ stomata at 75,000,000 ng/L (CeO_2_ NPs) under arsenic stress conditions. **(E, F)** showed the half closure of leaves’ stomata at 300,000,000 ng/L (CeO_2_ NPs) under arsenic stress conditions.

Our results indicated that As stress significantly (*p* ≤ 0.05) influenced the physiological attributes of Pak choi plants. Differing CeO_2_ NPs concentrations had an impact on the transpiration rate, net photosynthetic rate, stomatal conductance, and CO_2_ concentration, along with total chlorophyll and carotenoid levels as shown in **Table 3**. The CeO_2_ NPs foliar application (75,000,000 ng/L) showed a significant (*p* ≤ 0.05) increase in photosynthetic rate (77.38%) compared to the control group, followed by an increase of 54.40% and 38.03% at 150,000,000 ng/L CeO_2_ NPs and 300,000,000 ng/L CeO_2_ NPs, respectively. Similarly, a significant (*p* ≤ 0.05) difference in stomatal conductance (45.13%, 32.64%), transpiration rate (55.25%, 31.97%), and intercellular CO_2_ concentration (48.81%, 29.90%) were recorded at the level of 150,000,000 ng/L CeO_2_ NPs and 300,000,000 ng/L CeO_2_ NPs in comparison with control. Specifically, 75,000,000 ng/L CeO_2_ NPs were most effective in increasing stomatal conductance, transpiration rate, and CO_2_ concentration, with increases of 62.96%, 84.30%, and 58.87% compared to the respective control. CeO_2_ NPs foliar application showed improvement in Chl-a Chl-b contents with an increase of 67.58%, 71.42%; 47.98%, 53.97%; and 29.53%, 26.87% for 75,000,000 ng/L CeO_2_ NPs, 150,000,000 ng/L CeO_2_ NPs, and 300,000,000 ng/L CeO_2_ NPs, respectively, as compared to control. Similarly, in total chlorophyll and carotenoid levels, a maximum and significant (*p* ≤ 0.05) increase (69.15%, 62.01%) was observed at 75,000,000 ng/L CeO_2_ NPs, whereas 150,000,000 ng/L CeO_2_ NPs and 300,000,000 ng/L CeO_2_ NPs also had a significant improvement in total chlorophyll and carotenoid levels by 50.38%, 45.41%, and 28.43%, 23.46%, respectively, as compared to control as shown in **Figures 1**, **2**.

**Table 3:** Effect of CeO_2_ NPs on gas exchange parameters of Pak choi (*Brassica rapa* L. var. chinensis) grown under arsenic-contaminated soil.

Gas exchange parameters				
Treatments	Transpiration rate (mol m^−2^ s^−1^)	Photosynthetic rate (µmol m^−2^ s^−1^)	Stomata conductance (mol m^−2^ s^−1^)	CO_2_ concentration intercellular (µmol m^−2^ s^−1^)
NPs 0 (ng/L)	0.00081 ± 0.00056^c^	2.28 ± 1.18^c^	0.072 ± 0.0.33^c^	287.86 ± 39.36^c^
NPs 75,000,000 (ng/L)	0.00391 ± 0.0014^a^	4.04 ± 0.41^a^	0.117 ± 0.012^a^	373.87 ± 14.41^a^
NPs 150,000,000 (ng/L)	0.00205 ± 0.00041^ab^	3.52 ± 2.08^ab^	0.104 ± 0.016^ab^	348.02 ± 31.54^a^
NPs 300,000,000 (ng/L)	0.00122 ± 0.00029^b^	3.14 ± 2.22^b^	0.095 ± 0.054^b^	303.79 ± 30.37^b^

Values are the means ± standard deviation; different letters indicate significant difference among treatments at *p* ≤ 0.05.

### 3.3 Effect of CeO_2_ NPs (foliar) on oxidants and antioxidant enzyme activities in Pak choi plants under As stress

**Figure 3:**
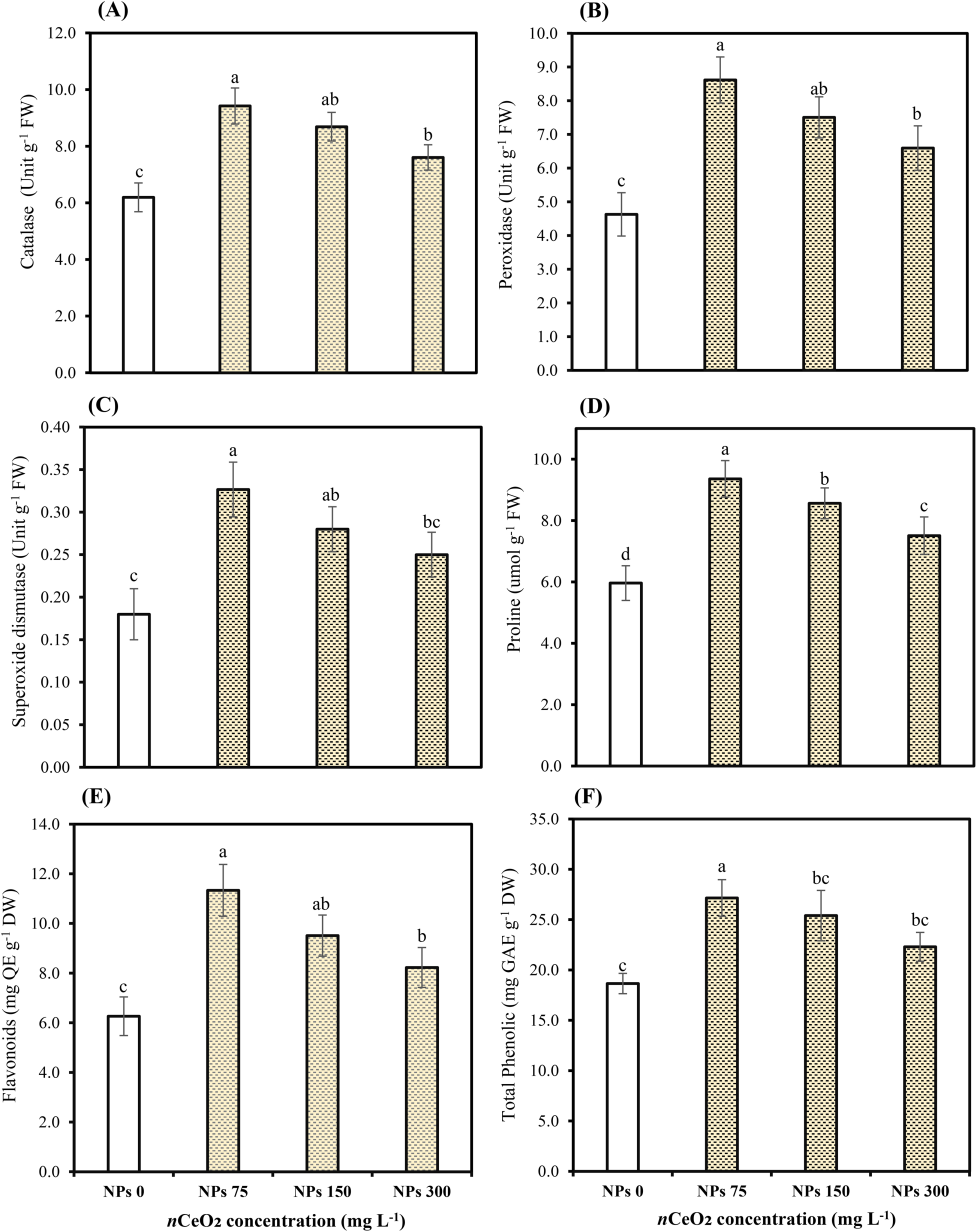
Effect of CeO_2_ NPs on catalase **(A)**, peroxidase **(B)**, superoxide dismutase **(C)**, proline **(D)**, flavonoids **(E)**, and total phenolic **(F)** in leaves of Pak choi (*Brassica rapa* L. var. chinensis) grown under arsenic-contaminated soil. Data are presented as the mean of three replicates ± standard deviation (SD). Different lowercase letters indicate significant/non-significant differences among treatments according to Tukey’s test (*p* ≤ 0.05).

**Figure 4:**
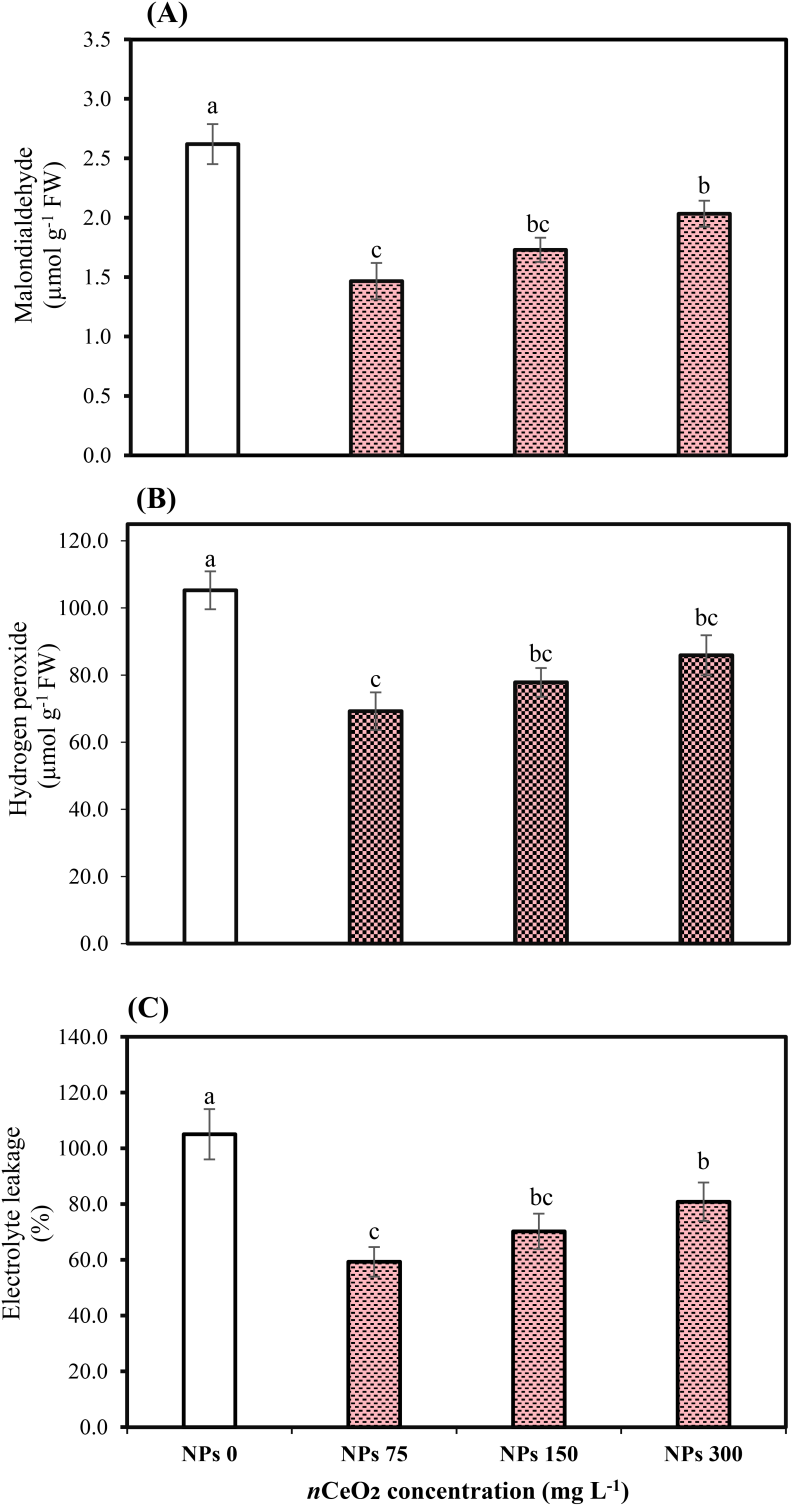
Effect of CeO_2_ NPs on malondialdehyde **(A)**, hydrogen peroxide **(B)**, and electrolyte leakage **(C)** in leaves of Pak choi (*Brassica rapa* L. var. chinensis) grown under arsenic-contaminated soil. Data are presented as the mean of three replicates ± standard deviation (SD). Different lowercase letters indicate significant/non-significant differences among treatments according to Tukey’s test (*p* ≤ 0.05).

The applied concentrations of CeO_2_ NPs significantly (*p* ≤ 0.05) affected the peroxidase (POD), catalase (CAT), and superoxidase dismutase (SOD) in the leaves of Pak choi plants grown in an As-contaminated environment. The results revealed that As stress considerably reduces antioxidant enzymatic activities, while the application of CeO_2_ NPs considerably enhanced the activities of antioxidant enzymes, as shown in **Figures 3** and **4**. The results indicate that CeO_2_ NPs at higher doses (150,000,000 ng/L and 300,000,000 ng/L) exhibited the lowest increase in antioxidant enzymes CAT and POD activities, with increments of 40.23% and 22.75%, and 62.13% and 42.47%, respectively. The maximum increment of antioxidant enzymatic activities was noted in the plant leaves with a lower dose of CeO_2_ NPs (75,000,000 ng/L). The improvement in CAT and POD activities was about 52.07% and 86.10% over the control. Similarly, in terms of SOD, a higher level (81.48%) was observed under 75,000,000 ng/L CeO_2_ NPs followed by 55.55% at 150,000,000 ng/L CeO_2_ NPs and 38.88% at 300,000,000 ng/L CeO_2_ NPs; all CeO_2_ NPs treatments were compared with the control. Higher hydrogen peroxide (H_2_O_2_), electrolyte leakage (EL), and MDA contents were observed in stressed control, while the lowest level was observed under 75,000,000 ng/L CeO_2_ NPs. The significant reduction in EL, H_2_O_2_, and MDA by 23.03%, 18.39%, and 22.39%, respectively, was examined in the treatments where 300,000,000 ng/L CeO_2_ NPs was applied, followed by 150,000,000 ng/L CeO_2_ NPs (33.13%, 26.06%, and 33.96%, respectively). On the other hand, 75,000,000 ng/L CeO_2_ NPs reduced the EL, H_2_O_2_, and MDA contents in Pak choi by 43.53%, 34.20%, and 44.02%, respectively, by comparing with control.

### 3.4 Effect of CeO_2_ NPs (foliar) on non-antioxidant enzyme activities in Pak choi plants under As stress

Among the foliar of CeO_2_ NPs, lower-concentration CeO_2_ NPs (75,000,000 ng/L) showed the most significant (*p* ≤ 0.05) increment in proline contents (13.52%). Proline content was 9.83% and 5.82% for leaves treated with 150,000,000 ng/L and 300,000,000 ng/L of CeO_2_ NPs in contrast to the control. On the other hand, the total phenolic content in Pak choi with the lower concentration (36.16% and 19.53%) was observed under both higher levels of CeO_2_ NPs (150,000,000 ng/L and 300,000,000 ng/L) as compared with 45.55% under lower levels of CeO_2_ NPs (75,000,000 ng/L). Furthermore, results revealed that the application of CeO_2_ NPs at both higher levels of 150,000,000 ng/L and 300,000,000 ng/L showed lower flavonoid activity by 21.78% and 5.36%, respectively. However, the maximum increase in flavonoid activity was observed under lower doses of CeO_2_ NPs (75,000,000 ng/L) in leaves, which was 45.13% under As stress, compared with stressed control as shown in **Figure 3**.

### 3.5 Effect of foliar application of CeO_2_ NPs on As in Pak choi plants under As stress

**Figure 5:**
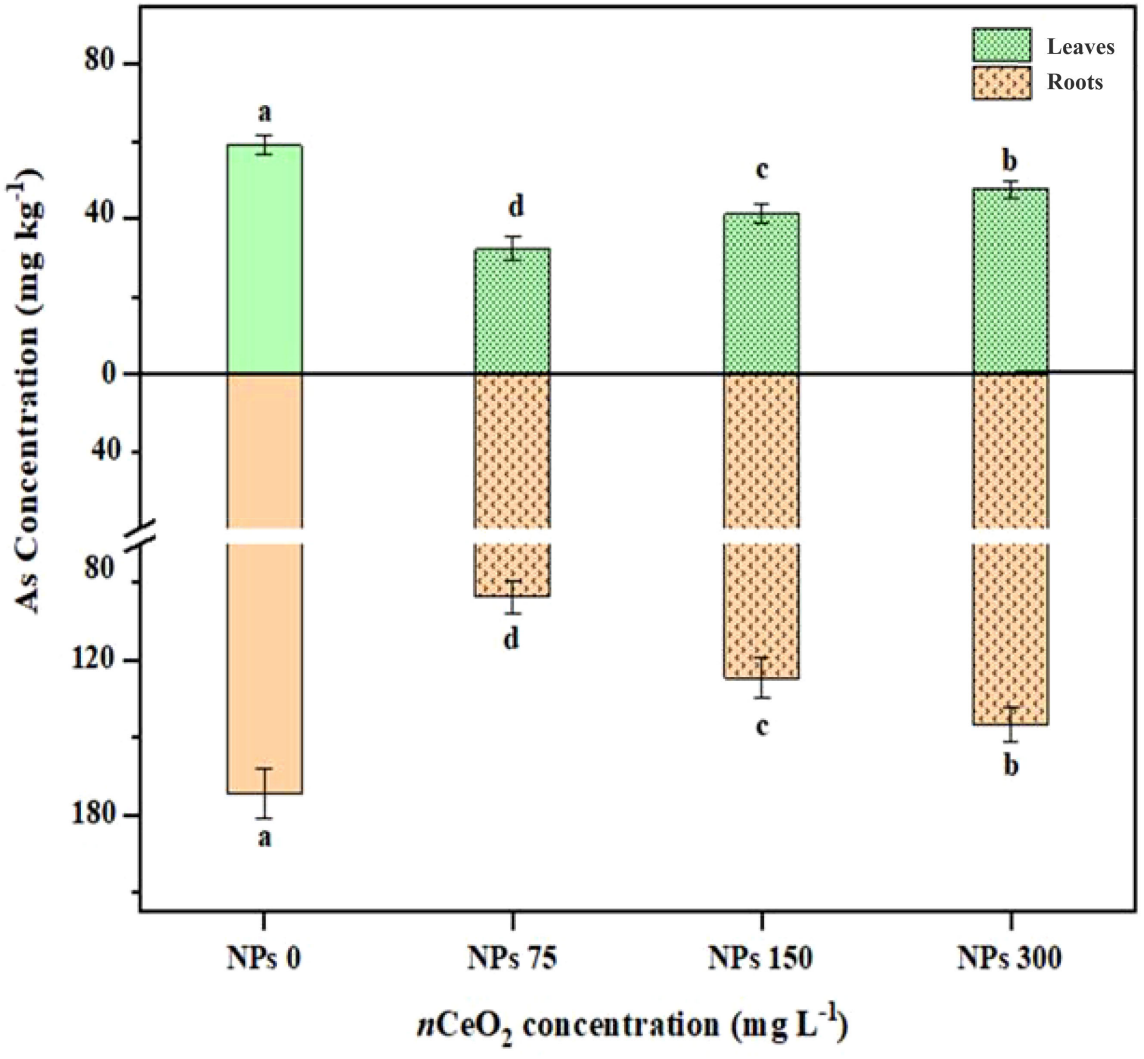
Effect of CeO_2_ NPs on arsenic (As) content in shoots and roots of Pak choi (*Brassica rapa* L. var. chinensis) grown under arsenic-contaminated soil. Data are presented as the mean of three replicates ± standard deviation (SD). Different lowercase letters indicate significant/non-significant differences among treatments according to Tukey’s test (*p* ≤ 0.05).

The data related to As concentration in different parts of Pak choi plants, including roots and shoots, are presented in **Figure 5**. The foliar application CeO_2_ NPs at different levels (75,000,000 ng/L, 150,000,000 ng/L, and 300,000,000 ng/L) minimized the As accumulation in root and shoot tissues. The 75,000,000 ng/L CeO_2_ NPs treatment showed a significant (*p* ≤ 0.05) decrease (20.89%) in As accumulation in root tissues compared to the control, with a percent decrease of 11.63% and 6.60% in 150,000,000 ng/L CeO_2_ NPs and 300,000,000 ng/L CeO_2_ NPs treatments, respectively. Similarly, 75,000,000 ng/L CeO_2_ NPs showed a significant (*p* ≤ 0.05) reduction in shoot in As accumulation by 45.11% followed by 150,000,000 ng/L CeO_2_ NPs with 30.27% and 300,000,000 ng/L CeO_2_ NPs with 19.33%.

### 3.6 Effect of CeO_2_ NPs on the root, shoot, and leaves in Pak choi plants under arsenic stress

Ce contents in Pak choi showed a dose-dependent trend, increasing with higher exposure concentrations under both treatments. The lowest concentration of Ce (59.69%) in the root portion was recorded under applied 75,000,000 ng/L CeO_2_ NPs. On the other hand, at higher levels of CeO_2_ NPs (150,000,000 ng/L and 300,000,000 ng/L), the accumulation in roots was 106.61% and 168.81%, respectively. The lowest Ce concentrations in shoots (542.14%) were recorded, where a lower dose of CeO_2_ NPs (75,000,000 ng/L) was applied under As stress, and higher values (1,980.31% and 3,144.91%) of Ce were observed where higher concentrations of CeO_2_ NPs (150,000,000 ng/L and 300,000,000 ng/L) were applied. Among the applied treatments, 75,000,000 ng/L CeO_2_ NPs showed minimum Ce contents by 1,763.76%, while Ce concentration increased under As stress conditions compared to control with an increase of 2,894.76% for 150,000,000 ng/L CeO_2_ NPs and 5,453.42% for 300,000,000 ng/L CeO_2_ NPs.

### 3.7 Effect of foliar application of CeO_2_ NPs on macronutrients in Pak choi plants under As stress

The applied treatments significantly (*p* ≤ 0.05) affected the nutrient contents of Pak choi under As stress environment. The nutrient concentration was decreased with the higher level of CeO_2_ NPs treatments. Among the treatments, 75,000,000 ng/L CeO_2_ NPs showed the most significant increase in Ca and K, 61.16% and 84.91%, 85% and 73.32%, and 87.77% and 72.95 for leaves, shoot, and root as compared to control treatment, respectively. On the other hand, plants treated with higher levels of CeO_2_ NPs (300,000,000 ng/L) showed a decreased concentration of Ca in leaves (22.76%), shoot (25.42%), and roots (45.78%) along with a lower concertation of K^+^ for leaves (31.18%), shoot (33.2%), and roots (31.62%), while a better response was observed under 150,000,000 ng/L CeO_2_ NPs by comparing with 300,000,000 ng/L CeO_2_ NPs in Ca and K^+^ of leaves (43.99% and 54.74%), shoot (55.72% and 49.51%), and roots (73.63% and 43.65%), respectively. A similar trend in Mg content was observed in Pak choi root, shoot, and leaves at 150,000,000 ng/L CeO_2_ NPs, and 300,000,000 ng/L CeO_2_ NPs showed decreased concentration of Mg in roots (51.12% and 25.83%), shoots (48.56% and 22.06%), and leaves (50.43% and 21.54%), respectively. In the case of 75,000,000 ng/L CeO_2_ NPs, plants showed better response in Mg concentration of roots (87.88%), shoot (79.41%), and leaves (76.83%) as compared to other treatments (**Table 4**).

**Table 4:** Effect of CeO_2_ NPs on cerium content and micro- and macronutrients in different parts (leaves, shoots, and roots) of Pak choi (*Brassica rapa* L. var. chinensis) grown under arsenic-contaminated soil.

Leaves (mg kg^−1^ DW)								
Treatments	Ca	Mg	Fe	Cu	Zn	K	Mn	Ce
NPs 0 (ng/L)	2,655.53 ± 190.72^c^	318.30 ± 25.84^c^	6.59 ± 7.13^c^	3.74 ± 0.36^c^	2.07 ± 0.25^c^	1,331.92 ± 114.68^d^	3.57 ± 0.43^c^	2.68 ± 0.13^d^
NPs 75,000,000 (ng/L)	4,279.83 ± 299.33^a^	562.85 ± 54.45^a^	20.87 ± 13.20^a^	5.88 ± 0.42^a^	3.75 ± 0.31^a^	2,462.98 ± 101.76^a^	6.02 ± 0.25^a^	49.89 ± 3.59^c^
NPs 150,000,000 (ng/L)	3,823.91± 269.49^ab^	478.83 ± 47.38^ab^	14.22 ± 7.10^a^	5.12 ± 0.32^ab^	3.29 ± 0.23^ab^	2,061.05 ± 126.51^b^	5.07 ± 0.23^b^	80.16 ± 6.06^b^
NPs 300,000,000 (ng/L)	3,260.14 ± 191.38^bc^	386.86 ± 30.31^bc^	11.64 ± 8.07^b^	4.38 ± 0.26^bc^	2.57 ± 0.31^bc^	1,747.23 ± 120.72^c^	4.31 ± 0.32^bc^	148.65 ± 3.74^a^
Shoots (mg kg^-1^ DW)								
NPs 0 (ng/L)	1,366.91 ± 110.15^d^	240.43 ± 21.75^c^	8.64 ± 1.08^c^	2.23 ± 0.25^c^	1.71 ± 0.21^b^	782.55 ± 147.07c	2.36 ± 0.18^c^	2.52 ± 0.53^d^
NPs 75,000,000 (ng/L)	2,535.16 ± 166.25 ^a^	430.37 ± 41.34^a^	18.19 ± 1.76^a^	3.83 ± 0.18^a^	3.17 ± 0.24^a^	1,356.38 ± 112.97a	4.17 ± 0.21^a^	16.20 ± 1.49^c^
NPs 150,000,000 (ng/L)	2,128.56 ± 132.44^b^	357.19 ± 23.72^ab^	13.74 ± 1.85^ab^	3.34 ± 0.19^a^	2.79 ± 0.75^a^	1,170.07 ± 111.44ab	3.51 ± 0.30^b^	52.49 ± 3.25^b^
NPs 300,000,000 (ng/L)	1,714.44 ± 106.33^c^	293.49 ± 24.72^bc^	10.58 ± 0.89^bc^	2.77 ± 0.21^b^	2.03 ± 0.25^b^	1,042.39 ± 75.21bc	2.81 ± 0.25^c^	81.88 ± 4.75^a^
Roots (mg kg^-1^ DW)								
NPs 0 (ng/L)	559.60 ± 56.43^c^	99.73 ± 9.75^c^	3.57 ± 0.76^b^	1.57 ± 0.23^b^	0.71 ± 0.13^b^	356.93 ± 20.87^c^	2.14 ± 0.27^c^	3.73 ± 0.35^d^
NPs 75,000,000 (ng/L)	1,050.73 ± 60.21^a^	147.14 ± 10.85^a^	12.22 ± 0.93^a^	2.83 ± 0.22^a^	1.31 ± 0.15^a^	617.32 ± 38.98^a^	3.69 ± 0.20^a^	5.96 ± 0.40^c^
NPs 150,000,000 (ng/L)	971.64 ± 58.02^a^	136.61 ± 18.49^b^	9.51 ± 0.64^a^	2.60 ± 0.17^a^	1.14 ± 0.10^a^	512.74 ± 36.39^b^	3.11 ± 0.15^ab^	7.71 ± 0.42^b^
NPs 300,000,000 (ng/L)	815.83 ± 50.79^b^	129.54 ± 13.67^bc^	7.17 ± 0.68^b^	2.26 ± 0.29^a^	1.06 ± 0.12^a^	469.82 ± 27.56^b^	2.73 ± 0.20^b^	10.03 ± 0.33^a^

Values are the means ± standard deviation; different letters indicate significant difference among treatments at *p* ≤ 0.05.

### 3.8 Effect of foliar application of CeO_2_ NPs on micronutrients in Pak choi plants under arsenic stress

Variable CeO_2_ NPs levels showed significant (*p* ≤ 0.05) effects on some micronutrients, which varied from one element to another. The Cu content in roots, shoots, and leaves under As stress was enhanced by 79.87%, 71.52%, and 56.99% under CeO_2_ NP-75 followed by higher doses of CeO_2_ NPs (150,000,000 ng/L and 300,000,000 ng/L) in roots (65.04% and 43.85%), in shoots (49.92% and 24.36%), and in leaves (36.68% and 17.08%), respectively, as compared to non-treated plants. The maximum Zn level in roots (84.11%), shoots (85.74%), and leaves (81.02%) were observed at 75,000,000 ng/L CeO_2_ NPs, while at a higher concentration of CeO_2_ NPs (150,000,000 ng/L and 300,000,000 ng/L), the minimum values were recorded in roots (60.04% and 48.13%), shoots (63.28% and 18.94%), and leaves (58.84% and 24.11%), respectively. Similarly, for root Mn contents, 150,000,000 ng/L and 300,000,000 ng/L CeO_2_ NPs resulted in a minimum concentration (45.55% and 27.61%) followed by the higher Mn concentration (70.04%) observed under the 75,000,000 ng/L CeO_2_ NPs. A similar trend was observed for shoots (76.55%, 48.58%, and 18.92%) and leaves (68.37%,41.84%, and 20.61%) under 75,000,000 ng/L, 150,000,000 ng/L, and 300,000,000 ng/L CeO_2_ NPs, respectively, in comparison with control. Results regarding Fe uptake through roots, shoots, and leaves are demonstrated in **Table 2**. Fe concentration was significantly (*p* ≤ 0.05) higher in roots (85.95%), shoots (64.78%), and leaves (88.04%) recorded at CeO_2_ NPs (75,000,000 ng/L). The CeO_2_ NPs decreased Fe concentration in roots, shoots, and leaves under higher concentrations. Individually, a significant (*p* ≤ 0.05) reduction in Fe concentration (59.83%, 43.77%, and 75.62%, and 24.34%, 25.22%, and 38.85%) was found in roots, shoots, and leaves, respectively, at 150,000,000 ng/L and 300,000,000 ng/L (**Table 4**).

## 4 Discussion

Over the past few decades, arsenic (As) contamination in soil has been a major concern due to prevalent toxicity, non-biodegradation, and widespread availability. In addition, As toxicity induced the production of ROS, leading to oxidative injuries inside cells, ultimately reducing growth and biomass accumulation. Many studies have reported that arsenic (As) exposure negatively affects plants at biochemical and molecular levels, influencing most physiological responses, including inhibition in overall growth processes, photosynthetic efficiency, and biomass accumulation (Kofroňová et al., 2020). Recently, nanotechnology has emerged as an innovative and eco-friendly approach to alleviate arsenic toxicity in plants. Among various NPs, CeO_2_ NPs hold significant potential as effective ROS scavenging properties. However, their effectiveness in mitigating As toxicity is insufficiently investigated (Anand and Pandey, 2024). While much attention has been given to As toxicity in grain crops, it is equally important to address its impact on leafy vegetables, which tend to accumulate higher levels of minerals and metals than grain crops.

The current study intended to investigate the impact of foliar application of CeO_2_ NPs in alleviating the effects of As stress on Pak choi. Results depicted that As stress negatively impacts plant morphology and physiological traits, resulting in the lower accumulation of nutrients in Pak choi plants. Previous studies by Skiba and Wolf (2019) and Lizzi et al. (2021) have demonstrated the beneficial effects of CeO_2_ NPs in improving nutrient retention, leading to notable improvements in plant height and overall biomass. Similarly, Gui et al. (2023) found that CeO_2_ NPs enhance plant growth and biomass production in sunflower plants under chromium stress. This can be associated with the potential of CeO_2_ NPs to reduce oxidative stress and improve antioxidant enzymatic activities.

In our study, As stress reduced chlorophyll and carotenoid contents along with a significant reduction in gas exchange attributes in Pak choi plants. However, CeO_2_ NPs particularly at lower concentrations significantly enhanced chlorophyll levels and photosynthetic efficiency showing more pronounced effects. Previous research supports that CeO_2_ NPs can greatly boost various growth indices in plants exposed to HM stress, including plant height, biomass, and chlorophyll content. For example, sunflower plants treated with 150,000,000 ng/L CeO_2_ NPs showed increased levels of chlorophyll a and b, which improved photosynthetic efficiency (Ma et al., 2022). In another study, Abbas et al. (2020) found that CeO_2_ NPs enhanced wheat growth by improving stomatal conductance, transpiration, and photosynthetic pigments. These NPs with their distinctive properties and resemblance with antioxidative enzymes may contribute to increased photosynthesis and other physiological processes. They act as catalysts and protect the structural integrity of chloroplast from damage (Jahani et al., 2019). On the other hand, we observed that higher concentrations of CeO_2_ NPs (150,000,000 and 300,000,000 ng/L) adversely affected plant growth and photosynthetic processes. It is well documented that higher CeO_2_ NPs (10,000 μg/L) may inhibit photosynthesis as higher concentration CeO_2_ NPs can hinder electron transport in the PSII electronic pathway leading to decreased efficiency of this system (Kamali-Andani et al., 2022). Our results align with Agathokleous (2021), who reported that under lower concentrations of CeO_2_ NPs, chl a and b were increased but decreased at higher concentrations.

Plants continuously generate ROS under stressful circumstances and trigger oxidative stress, adversely affecting several cell components. The present study highlighted that lower concentration treatments of CeO_2_ NPs reduced electrolyte leakage in Pak choi, indicating improved membrane integrity and stress resistance. These results are consistent with those of Salehi et al. (2018) and Zhang et al. (2019), in which foliar-applied CeO_2_ NPs decreased electrolyte leakage in leaves of spinach and *Phaseolus vulgaris* L. Plants activate enzymatic defense mechanisms, including SOD, POD, and CAT to reduce the harmful effects of ROS. Our results showed that As stress lowered the activities of antioxidant enzymes; however, foliar applied CeO_2_ NPs at an optimum concentration (75,000,000 ng/L) increased antioxidant enzyme activities. This effect is ascribed to the capacity of CeO_2_ NPs to emulate enzymatic functions, functioning as both oxidants and antioxidants, with a notable protective role associated with their ability to replicate the activity of SOD (Hussain et al., 2017). Salehi et al. (2021) and Haghmadad Milani et al. (2023) reported that a concentration of 100 mg/L CeO_2_ NPs had negative effects on certain physiological parameters of plants. However, the observed variations are likely influenced by other factors like plant development conditions, specific plant species, application methods, and the concentration and duration of exposure (Skiba et al., 2021). This also underscores the importance of carefully considering the concentration of CeO_2_ NPs used. Furthermore, in another study, CeO_2_ NPs significantly enhanced antioxidant enzymes and proline levels in stressed plants (Imani et al., 2023). Our results indicate that total phenol was reduced under As stress and higher doses of CeO_2_ NPs. A study found that CeO_2_ NPs increased total phenols in soybeans and decreased starch, demonstrating that NPs affect nutritional quality in a species-specific manner (Gui et al., 2023). The results of the present study found that increasing concentrations of CeO_2_ NPs reduced the levels of essential nutrients in the roots and leaves of Pak choi, with a significant decline at the higher concentration (300,000,000 ng/L) compared to the optimal CeO_2_ NPs (75,000,000 ng/L) concentration under As stress conditions. CeO_2_ NPs can enhance the uptake of essential nutrients, such as Ca^2+^ and Mg^2+^, crucial for maintaining ion homeostasis in plants under stress. This improved nutrient balance helps reduce the negative effects of As on plant growth (Haghmadad Milani et al., 2023). Ayub et al. (2023) found that CeO_2_ NPs can alleviate cadmium toxicity in maize (*Zea mays*) by enhancing growth and nutrient content. As a result, there was an increase in biomass and an improvement in nutrient profiles, including higher levels of zinc (Zn) and iron (Fe). The increase in essential micronutrients is attributed to the ability of NPs to enhance antioxidant defenses and reduce oxidative stress. On the other hand, in our results, a decrease in nutrient concentration at higher concentrations of NPs (300,000,000 ng/L) can be linked to the hindrance of nutrient absorption and delivery of essential nutrients, affecting the nutritional balance in plants and leading to deficiencies or toxicities. In addition, elevated NP concentrations may physically hinder the plant’s nutrition and water transport systems, obstructing the passage of chemicals within the plant (Hong et al., 2021). Skiba and Wolf (2019) concluded that CeO_2_ NPs applied at 200 ppm level decreased the Fe, Cu, and Zn uptake in roots and shoots. This study aligns with the present study where a higher concentration of CeO_2_ NPs (300,000,000 ng/L) also decreased the Fe, Zn, and Cu in leaves, roots, and shoots of the Pak choi plant.

In addition to its biological role in the bioaccumulation of CeO_2_ NPs in plant tissues, it significantly contributes to the reduction of HM adsorption on the root surfaces of plants (Anand and Pandey, 2024). Research has demonstrated that CeO_2_ NPs can reduce the uptake and translocation of HMs within plant tissues (Liu et al., 2014). Ayub et al. (2023) also demonstrated that CeO_2_ NPs help plants manage HM stress by decreasing Cd absorption and translocation in maize. Furthermore, Wang et al. (2024) reported that CeO_2_ NPs (100 mg/L) did not affect the accumulation of As in rice. This might happen due to the formation of aggregates and the selected concentration of CeO_2_ NPs. However, NPs inhibit As absorption through distinct mechanisms such as *in vitro* adsorption to reduce uptake by plant roots (Khan et al., 2024). This adsorption prevents excessive As from being accumulated in the plant (Yang et al., 2022). Additionally, NPs promote plant growth and regulate not only the anti-oxidative defense system but also *in vivo* complexation to restrict As translocation to the aboveground portion of the plant, thereby limiting its uptake (Ghorbani et al., 2024).

In summary, we have observed that CeO_2_ NPs at lower concentrations (75,000,000 ng/L) are beneficial for Pak choi growth and nutritional quality under As stress, which suggests that these NPs at optimum concentration could be used as effective agrochemical strategies to improve plant growth development and nutrient accumulation while reducing As toxicity. However, it is critical to recognize that higher concentrations of CeO_2_ NPs induce phytotoxicity including inhibited growth and oxidative damage. This emphasizes the optimization of NPs before application to avoid adverse effects on plant and human health. The optimal concentration of NPs is critical for mitigating the accumulation of toxic metals in edible parts of crops, thus ensuring food safety and security.

## 5 Conclusion

The current study demonstrated the potential impacts of CeO_2_ NPs via foliar application in mitigating As stress in Pak choi. Our findings indicate that CeO_2_ NPs considerably enhanced Pak choi growth and biomass by improving morphological and physiological traits. Lower concentrations (75,000,000 ng/L) of CeO_2_ NPs showed a significant reduction in As toxicity in plants, resulting in enhanced plant growth, photosynthetic rate, and antioxidant enzyme activities (CAT, SOD, APX, and POD) by reducing oxidative stress. In contrast, higher concentration (300,000,000 ng/L) did not yield beneficial effects and caused phytotoxicity, highlighting the importance of NP optimization before application. This study found that lower CeO_2_ NPs effectively combat As stress, offering a promising solution to lower metal concentration and mitigate yield loss in Pak choi. This finding presents a new approach to applying the CeO_2_ NPs foliar method to enhance Pak choi cultivation in As-contaminated soil, which has significant application for food safety and crop quality. Further research is required to determine the optimum application rates for different crops and the long-term effects of these CeO_2_ NPs on the ecosystem. Future research should also focus on the mechanistic pathways of these NPs and the effect on the soil microbial community to evaluate broader ecological impact and ecosystem stability.

## Data Availability

The raw data supporting the conclusions of this article will be made available by the authors, without undue reservation.
